# Portuguese Family Physicians’ Perceptions on Pain Management—A Qualitative Study Protocol

**DOI:** 10.3390/ijerph192214792

**Published:** 2022-11-10

**Authors:** Joana Fernandes Ribeiro, Sofia Baptista, Marta Pinto, Ana Mendes, Hugo Almeida, Andreia Teixeira, Carlos Martins

**Affiliations:** 1 USF Faria Guimarães, ACeS Porto Oriental, 4200-292 Porto, Portugal; 2 Centre for Health Technology and Services Research (CINTESIS), University of Porto, 4200-450 Porto, Portugal; 3 ACeS Porto Ocidental, 4150-502 Porto, Portugal; 4 Department of Community Medicine, Information and Health Decision Sciences (MEDCIDS), Faculty of Medicine, University of Porto, 4200-450 Porto, Portugal; 5 Faculty of Medical Sciences, NOVA University of Lisbon, 1169-056 Lisbon, Portugal; 6 USF Porto Centro, ACeS Porto Oriental, 4000-478 Porto, Portugal; 7 USF Barão Nova Sintra, ACeS Porto Oriental, 4300-367 Porto, Portugal; 8 ADiT-LAB, Instituto Politécnico de Viana do Castelo, Rua Escola Industrial e Comercial Nun’Álvares, 4900-347 Viana do Castelo, Portugal; 9 H4A Primary Health Care Research Network, Porto, Portugal

**Keywords:** pain management, family physicians, medical education, focus groups

## Abstract

Pain is an important cause of disability and constitutes the main reason people seek medical care, especially in general practice. Nevertheless, nearly half of adult Europeans with chronic pain receive inadequate pain treatment. Limited knowledge about pain among physicians is recognized as a key barrier to treatment. This is due to the well-known insufficiency in pain education at both undergraduate and postgraduate levels. There is a scarcity of research exploring the perceptions of family medicine physicians on these issues. This study aims to evaluate the perceptions of these professionals concerning medical education, as well as their knowledge, skills, and preparedness to manage chronic pain and collect suggestions for improvement. A qualitative exploratory study will be performed using synchronous virtual focus groups and purposive sampling. Eligible participants will be 3rd- and 4th-year family medicine residents and family medicine specialists with at least five years of practice. Sample size and number of focus groups will depend on data saturation. A semi-structured guide will be used. A thematic categorical analysis will be conducted after verbatim transcription of the audiofiles. This protocol has been approved by the Health Ethics Committee.

## 1. Introduction

Pain is an important life-protecting system, but can interfere greatly with a person’s quality of life and general functioning; thus, it has major clinical, social and economic burdens [1,2,3,4,5]. It is the main reason that people seek medical care, especially in general practice [2]. According to the Global Burden of Disease 2016 and 2019, pain is itself—and along with pain-related diseases—a leading cause of disability and disease burden globally [3,6,7].

Defined as “an unpleasant sensory and emotional experience associated with, or resembling that associated with, actual or potential tissue damage” [8], pain is a personal experience that cannot solely be understood from activity in the sensory neurons [8]. This new definition from the International Association for the Study of Pain demonstrates the complexity of pain, and how challenging it can be for both patients and practitioners. Acute pain can be caused by injury, surgery, illness, trauma, or painful medical procedures, and serves as a warning sign of threat to the body [8,9]. It has the duration of the causing event, a mechanism that reinforces its protective role [8,9]. Differently, chronic pain results from a maladaptive process [8]. It lasts longer than three months [10], and involves irreversible pathophysiologic changes and loss of function [2], and therefore it is much more difficult to treat than acute pain. Moreover, it is devoid of purpose [10].

Chronic pain of moderate to severe intensity occurs in 19% of adult Europeans [1] with nearly half receiving inadequate pain management [1,5]. In Portugal, a landmark cross-sectional nationwide epidemiological study performed between 2007 and 2008, determined a prevalence of 36.7% for chronic pain [11], associated with high consumption of healthcare resources [12,13] and high economic impact [13,14]. Recently, a study to investigate the prevalence of pain in Portuguese primary health care found that more than three million people suffer from chronic pain in that setting [15]. Patients tend to visit a family doctor for pain treatment [2], and this process takes four years for the patient to receive a proper diagnosis. The National Program for Prevention and Pain Management of the Portuguese Directorate-General for Health recognizes the need for action and highlights the importance of equal access to proper pain management [13].

One of the most relevant ways to reduce the prevalence of chronic pain is to prevent acute pain from occurring, and managing it well when it does occur [16,17]. However, a lack of knowledge about pain among physicians is recognized as a key barrier to effective pain treatment and management [2,18,19,20]. This insufficiency in pain education, both undergraduate and postgraduate, is acknowledged by entities such as the International Association for the Study of Pain and the European Pain Federation [1,2,18,21]. The same reality applies to Portugal, which suffers from a lack of consistent educational programs [2,13,22]. The National Program for Prevention and Pain Management of the Portuguese Directorate-General for Health set the improvement of healthcare professionals pain education as a main goal to achieve for the period 2017–2020, but there is still a long road ahead.

In Portugal, it takes six years for a medical student to graduate. After that period, the resident physician enters a one-year program of professionalizing general training that grants them access to autonomous practice and specializing programs [23]. A family medicine residency consists of four years of specialization after the general year [24]. It comprises the following trainings: primary care setting; 3–9 months in women’s health, mental health and infantile/juvenile health; 576 h in urgent and emergency care settings; and 2–7 months plus 480 h of optional training (where the resident physician chooses what areas best suits one’s interests) [24]. There are also optional and obligatory courses to complement specific educational gaps [24]. The national residency program is divided into seven regional coordination groups which are responsible for operationalizing and supervising the program in each coordination and for organizing complementary courses [24]. Pain education is not formally present in the residency program. Residents can practice in a pain clinic or in a palliative care unit during their optional trainings or engage in pain courses outside residency, because there are no formal residency pain courses (optional or obligatory). There are various options of postgraduate courses, with different lengths being offered, either by medical schools or private enterprises.

Considering the privileged contact that family medicine physicians have with their populations, and therefore, their privileged position for pain management, it is important that these professionals have adequate skills to address it [2]. Some studies already show how physicians feel about their preparedness to treat pain [25], but there is however a scarcity of research exploring the perceptions and opinions of family medicine physicians on these issues. This is specifically the case concerning undergraduate and residency medical education on pain management.

With this study the authors aim to:

- Explore self-perceptions of family medicine residents and specialists concerning the knowledge and skills to manage chronic pain.
- Assess preparedness and confidence of family medicine residents and specialists to manage chronic pain.
- Explore perceptions of family medicine residents and specialists regarding undergraduate and residency medical education on chronic pain management.
- Collect recommendations for improvement on all different levels of medical education.

## 2. Materials and Methods

### Study Design and Methodological Framework

A qualitative exploratory study will be performed using focus groups and subsequent thematic categorical analysis to explore the perceptions and ideas of family medicine physicians on chronic pain management [26]. Qualitative methods will enable us to explore poorly documented fields and provide in-depth understanding of real-world problems, thus paving the way for more directed approaches [27,28]. These methods also enable researchers to study the experiences and perceptions of individuals [27], and how they make meaning from their experiences [29]. Focus groups are an increasingly popular approach in medical education and are particularly suitable for exploratory research, promoting the exchange of ideas and perceptions especially in poorly understood or unclear topics [29,30,31,32]. Promoting group discussion rather than solo interviews can generate insight/ideas and lead to a topic being pursued in greater depth [33]. The purpose of this study design is to gather residents’ and consultants’ perceptions, feelings, actions, and circumstances concerning chronic pain management in a primary care setting [29]. Focus groups have been previously used to study the views of family medicine physicians regarding pain and other thematics [25,30].

Participants’ general characterization will be collected using a questionnaire (Table 1). The proposed time for the study is available in Figure S1. The Consolidated Criteria for Reporting Qualitative research guideline will be used for adequate reporting of the study and protocol design [34].

## 3. Participants

Participants considered eligible will be 3rd- and 4th-year family medicine residents and family medicine specialists working in the Portuguese National Health Service. The authors decided to collect information from residents who have completed at least half of their residency and therefore have more experience to share. Family medicine specialists have at least five years of practice as specialists. There are no other inclusion or exclusion criteria.

The authors plan to use purposive sampling to select physicians that meet the inclusion criteria [31].

The Portuguese National Health Service is geographically divided into five continental Health Regional Administrations that have financial and administrative autonomy, and therefore differences regarding human resources management. The Azores and Madeira islands have their own Regional Health Services managed by the respective regional governments which are not part of the National Health Service.

With this in mind, there will be an attempt to recruit physicians from each of the Health Regional Administrations, plus Madeira and the Azores’ Regional Health Services, to reflect the diversity within the population under study and stimulate discussion with the aim providing new insights into the topic [29].

Physicians will be recruited through residents’ committees and regional administrations communication channels, social media, and peer networking with a brief overview of the project. Those willing to participate in the study will receive an email with more detailed information, a formal invitation for the focus group, and an informed consent form to sign in advance. Participants will be informed that their statements are confidential and will be kept on a password-secured computer and are only to be used for research. The final size of the sample will depend upon data saturation in order to ensure dependability [29,31].

### Focus Groups

Synchronous virtual focus groups will be the setting for data collection using a safe digital meeting platform. We believe that online meetings will simplify the process of gathering professionals from different geographical parts of the country. New and well-built online meeting platforms that provide a safe and less exposed environment and an easy way to keep records [29] have emerged in recent years [35]. We expect that the participants will have knowledge and easy access to technology, which guarantees equal access [35].

The main purpose of the focus group is to provide an environment to bring up different perspectives within the same group and understand physicians’ problems. Thus, we will facilitate debates where diversity and harmony are balanced, so everyone can share their point of view [30]. Each focus group will contain between six and ten participants, with an optimum number of eight participants [30]. This is because group sizes below six can make it difficult to sustain a meaningful discussion, and a group of >10 may prove difficult to manage [29,30,36]. Respecting the criteria of homogeneity, participants will be distributed according to their professional status, and thus there will be focus groups with family medicine residents and focus groups with family medicine specialists. Homogeneous groups on medical education research encourage a relatively uninhibited and consequently more balanced discussion [30].

Focus groups will be conducted by a moderator and an observer. These people are two family medicine residents with previous training in qualitative research and no established relationship with participants. The moderator will have the responsibility to guide the interview using a semi-structured guide previously prepared for the study based on a questioning route model of Krueger & Casey [37] and supported by scientific evidence. The guide has been revised by pain and qualitative methodology experts and will be tested with a pilot (Table 2). A non-directive moderator style will be used [29], respecting the interview guide but giving space for themes and issues brought up during the discussion. The observer will support the moderator while taking notes and being responsible for technical issues [29]. The meetings will be conducted in Portuguese. The authors plan to pursue data collection until data saturation is achieved and no new information is emerging. The number of focus groups will therefore be limited mainly by that factor.

## 4. Data Analysis

Data collection will include the audio files of the focus groups, field notes and participants’ general characterization data. Each participant and each focus group will be given an alphanumeric code to ensure confidentiality. The audio files of the focus groups will be transcribed and checked for accuracy by two family medicine residents.

Transcripts will then be analyzed inductively for emerging themes by two authors independently and supervised by an expert in qualitative research. Similar ideas will be clustered together considering outlier topics. Any divergencies shall be settled through debate and consensus and supervised by an expert throughout the process to guarantee coding consistency. In the end, participants will be provided with a copy of the transcripts to give them the opportunity to provide additional corrections or comments.

The most representative citations of each theme will be selected by the first author.

Transcripts will be kept on a personal computer belonging to one of the authors with no network connection. These data will be destroyed at most one month after data analysis.

The authors plan to employ some techniques to improve the study quality. Methodological and data triangulation, member checking as well as the involvement of different researchers in qualitative coding can ensure the reliability of the qualitative data. Field notes and a reflexivity diary will be used to refine the interview guide, as well as to identify codes and questions to explore.

## 5. Conclusions

This study intends to unveil the reality of chronic pain management in a primary care setting through the voice of the physician. The goal is to create a space for reflection on the needs, the opportunities, and challenges on pain management. Furthermore, the authors intend to create a space where physicians can have an active role in the development of systematized education in chronic pain. Considering the scarcity of the literature reflecting on these matters, the results are rather unpredictable. However, the authors anticipate this study can raise more awareness about pain inside the medical community. If the results indicate the need, then there is the intention to contribute to the design of a chronic pain medical training proposal for family medicine curricula.

## Figures and Tables

**Table 1:** General Characterization Questionnaire.

Questions	Answers
Gender	Male; Female; Other
Age	Free answer
Which RHA do you have professional contract with?	North RHA; Lisboa and Vale do Tejo RHA; Alentejo RHA; Algarve RHA; Azores RHD; Madeira RHD
Which Medical School have you graduated from?	Free answer
Did you ever experience or are experiencing now any chronic painful condition?	Yes; No
If your previews answer was YES, could you rate the level of pain you experienced or are experiencing?	NPRS (1–10)

RHA—Regional Health Administration; RHD—Regional Health Direction; NPRS—numeric pain rating scale.

**Table 2:** Interview Semi-Structured Guide.

**Opening questions**
Do you frequently have patients with uncontrolled pain? Did you have access to pain education during your medical graduation? Residency? Do you frequently need to send patients to pain consultation due to difficulty/incapability in managing their pain? Did it ever happen to have patients sent back?
**Introductory questions**
What do you consider to be the main obstacles in controlling the pain of your patients? What do you consider to be your main difficulties in managing your patients’ chronic pain? How do you deal with those difficulties? Have those difficulties led you to send patients to pain consultation? What is your opinion regarding the number of hours dedicated to pain education during undergraduate medical education? Residency? What suggestions do you have regarding pain education methods?
**Transition questions**
How do you evaluate your knowledge and skills concerning anamnesis, use of pain questionnaires, and physical exam in a patient with chronic pain? How would you evaluate your knowledge and skills concerning therapeutic initiation and management? Have you undergone any training outside residency in pain/pain management? Why? What sort of training/course? What do you think about family medicine physician’s role on keeping patient’s chronic pain under control?
**Key questions**
What do you think about pain education in family medicine? Would you suggest anything different? In your opinion what measures could improve pain management quality in primary care setting? What do you highlight as important to empower family medicine physicians to provide even better chronic pain management care?
**Ending questions**
Is there anything you would like to say that has not been covered?

## Supplementary Materials

The following supporting information can be downloaded at: https://www.mdpi.com/article/10.3390/ijerph192214792/s1, Figure S1: Study chronogram.
