# Supplementary material for: Portuguese Family Physicians’ Perceptions on Pain Management—A Qualitative Study Protocol
Source: Int J Environ Res Public Health. 2022 Nov 10;19(22):14792. doi: 10.3390/ijerph192214792 (PMC9691093; doi:10.3390/ijerph192214792)

FIGURE S1 – STUDY CHRONOGRAM

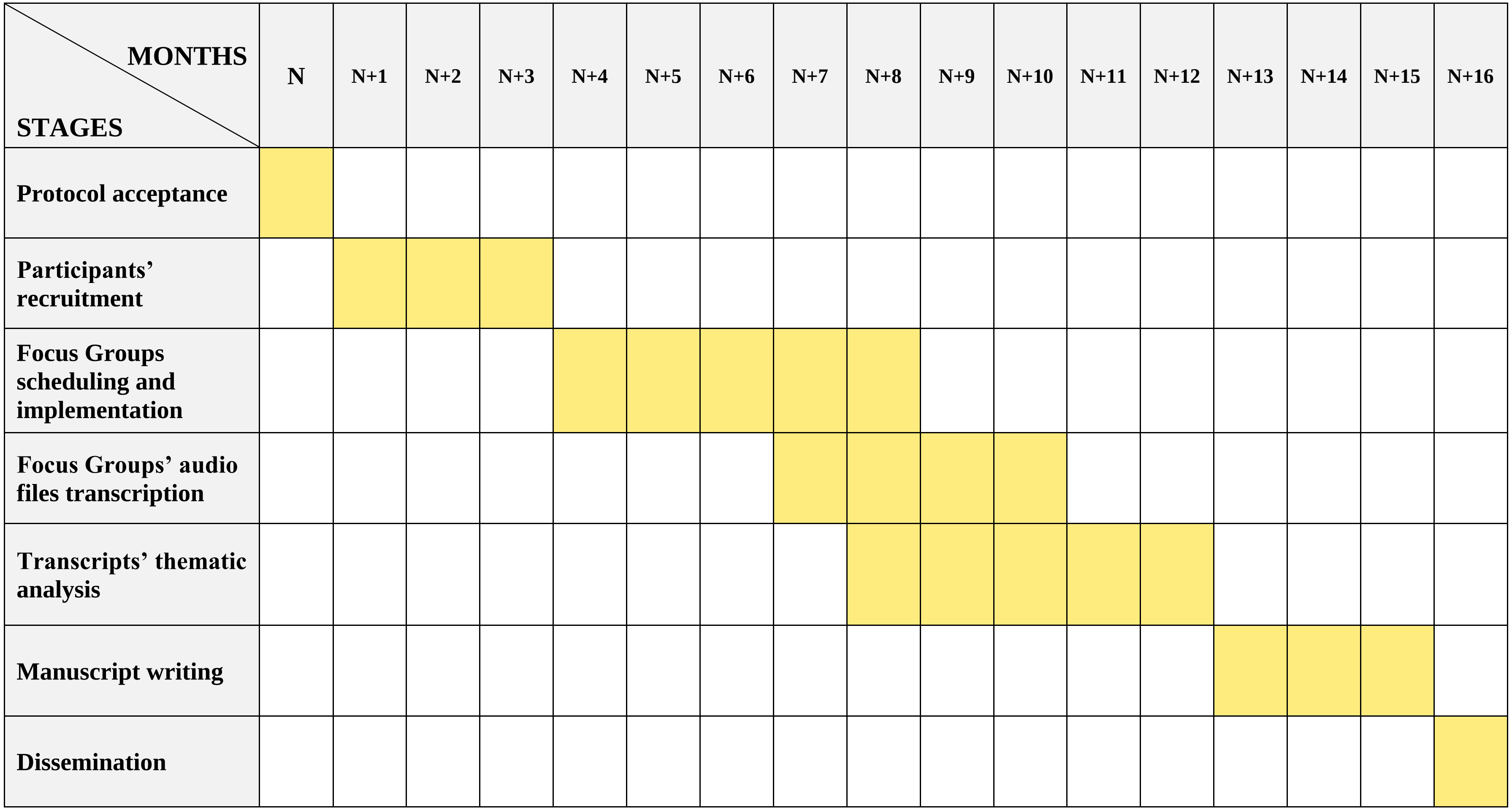

Supplement: Supplementary file 1 [file ijerph-1960135-supplementary.pdf › ijerph-1960135-supplementary.pdf]
